# Super Scan Caused by Parathyroid Carcinoma Observed Both in ^18^F-FDG PET/CT Scan and Tc-99m MDP Bone Scintigraphy

**DOI:** 10.4274/mirt.70188

**Published:** 2017-10-02

**Authors:** İsa Burak Güney, Semra Paydaş, Hüseyin Tuğsan Ballı

**Affiliations:** 1 Çukurova University Faculty of Medicine, Department of Nuclear Medicine, Adana, Turkey; 2 Çukurova University Faculty of Medicine, Department of Medical Oncology, Adana, Turkey; 3 Çukurova University Faculty of Medicine, Department of Radiology, Adana, Turkey

**Keywords:** Super scan, 18F-fluorodeoxyglucose positron emission tomography/computed tomography, parathyroid carcinoma

## Abstract

Super scan is a well-known finding described in skeletal scintigraphy characterized by uniform symmetrically increased radiopharmaceutical uptake by bones and consequently diminished renal parenchymal activity. Sy et al. hypothesized that the faint visualization of renal cortex in bone scintigraphy might be the result of increased uptake of radiopharmaceutical by pathologic bones and reduced phosphate excretion. The super scan on ^18^F-fluorodeoxyglucose positron emission tomography/computed tomography (^18^F-FDG PET/CT) has been observed in various conditions such as prostate cancer, lung cancer, renal adenocarcinoma, gastric cancer and primitive neuroectodermal tumor of the kidney. Herein we report the first case of super scan in a 68-year-old-woman with parathyroid carcinoma observed both in ^18^F-FDG PET/CT and Tc-99m methylene diphosphonate bone scintigraphy. There were extensive hypermetabolic lesions throughout the skeleton in ^18^F-FDG PET/CT. In contrast to the intense hypermetabolism of the skeleton; the liver, skeletal muscles of the limbs, mediastinum, bowel and especially the brain showed very low FDG uptake. Additionally, there was increased skeletal radiotracer uptake relative to soft tissue, and faint genitourinary tract activity in bone scintigraphy.

## Abstract (tr)

Süper görüntü kemik sintigrafisinde iyi bilinen bir bulgu olup simetrik olarak artmış kemik tutulumu ve böbreklerin görüntülenmemesi ile karakterizedir. Sy ve ark. hastalıklı kemik tarafından artan radyofarmasötik alımının azalmış fosfat atılımına ve kemik sintigrafisinde böbreklerin çok düşük düzeyde görüntülenmesine neden olduğunu ileri sürmüşlerdir. ^18^F-florodeoksiglukoz pozitron emisyon tomografisi/bilgisayarlı tomografide (^18^F-FDG PET/BT) süper görüntü, prostat kanseri, akciğer kanseri, böbrek adenokarsinomu, gastrik kanser ve böbreğin ilkel nöroektodermal tümörü gibi çeşitli kanserlerde rapor edilmiştir. Burada ilk kez paratiroid karsinoması olan 68 yaşındaki bir kadında hem ^18^F-FDG PET/BT, hem de Tc-99m metilen difosfonat kemik sintigrafisinde süper görüntü bulgularını sunduk. ^18^F-FDG PET/BT’de tüm iskelet sisteminde geniş hipermetabolik lezyonlar vardı. İskelet sistemindeki hipermetabolizmanın aksine, karaciğerde, interkostal kaslarda, mediastinumda, barsaklarda ve özellikle beyinde FDG tutulumu çok düşük seviyedeydi. Ek olarak, kemik sintigrafisinde yumuşak dokuya nazaran iskelet sisteminde artmış radyofarmasötik tutulumu ve zayıf genitoüriner sistem aktivitesi vardı.

## Figures and Tables

**Figure 1:**
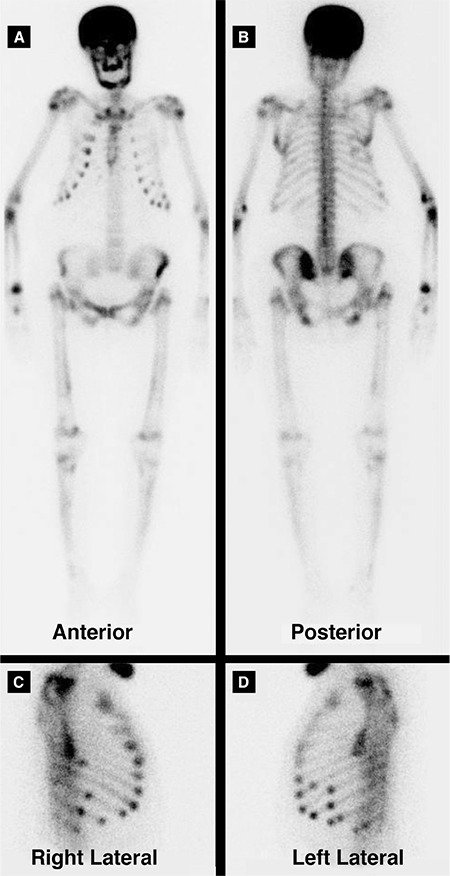
Tc-99m MDP bone scintigraphy. A 68-year-old women was admitted to the hospital on October 2013 with a mass on the cervical region. An excisional biopsy revealed parathyroid cancer. PET/CT showed multiple hypermetabolic lesions in the skeleton. The patient was treated by combination chemotherapy (including cisplatin and etoposide) plus zoledronic acid with good clinical response. Two years later, she presented with fatigue. Her laboratory results showed severe hypercalcemia and renal failure (serum calcium level 16.6 mg/dL, blood urea nitrogen/creatinine: 32/2.3 mg/dL). The parathormone level was 1859.6 pg/mL. Hydration was performed, calcitonin was prescribed but the hypercalcemia could not be controlled. Tc-99m MDP bone scintigraphy revealed intense radiotracer uptake involving almost the entire skeleton with high bone to soft tissue uptake (A and B). Bilateral kidneys were almost invisible (A and B). These findings are suggestive of ‘metabolic superscan’. There was focal radiotracer uptake in several ribs considered as Brown tumors according to CT findings (C and D). Brown tumors represent a reparative cellular process rather than a neoplastic process (1). The increased uptake of radiopharmaceutical by pathologic bone results in reduced phosphate excretion, thereby producing faint renal images on bone scintigraphy. Wiegemann et al. (2) reported that bone resorption, alkaline phosphatase level, and parathyroid hormone activity were not correlated with Tc-99m pyrophosphate uptake by the bone. Malignancies with known super scan in bone scintigraphy include prostate cancer, breast cancer, lung cancer, lymphoma, urinary tract transitional cell carcinoma, nasopharyngeal carcinoma and carcinoma of the colon (3,4,5). Hematologic conditions like leukemia, lymphoma, myelofibrosis, Waldenstrom’s macroglobulinemia have also been reported to be associated with super scan. This feature can also be detected in metabolic bone diseases like renal osteodystrophy, Paget’s disease and hyperparathyroidism (2)

**Figure 2:**
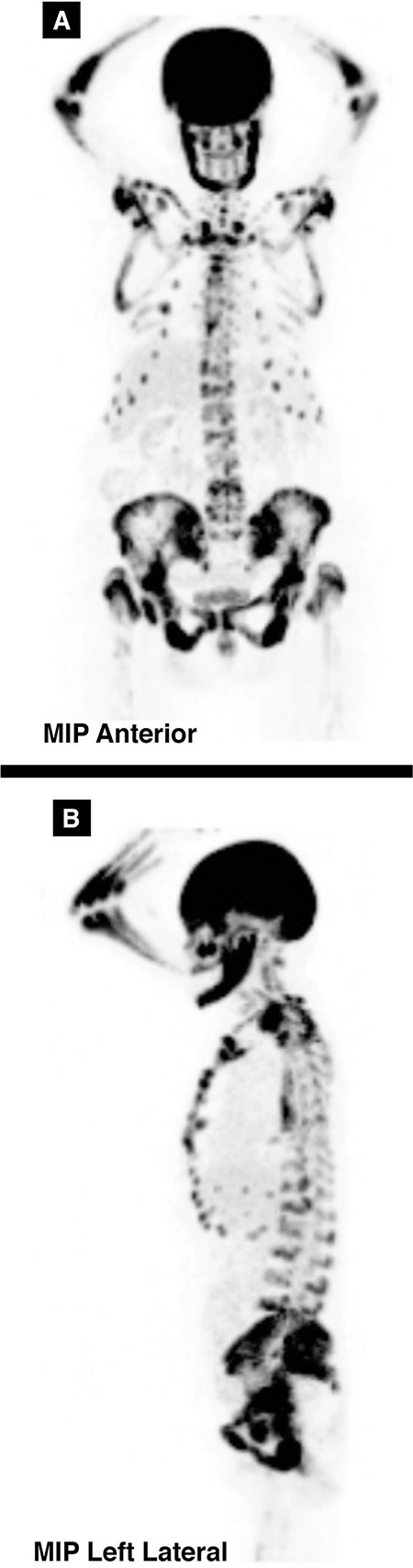
^18^F-FDG PET/CT demonstrated diffuse and intense hypermetabolism throughout the skeleton (A and B). In FDG PET scan, the faint visualization of the brain, renal cortex, and soft tissue might be the result of extraordinarily high uptake of FDG by skeletal lesions. The presented patient was not on any medications that could have disturbed cerebral glucose metabolism, such as corticosteroids or sedatives

**Figure 3:**
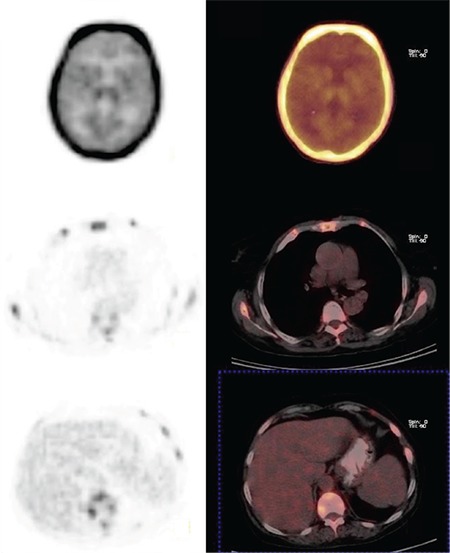
Interestingly the mediastinum, skeletal muscles of the limbs, bowel and especially the brain showed very low FDG uptake on ^18^F-FDG PET/CT. A parathyroid adenoma failed to be detected by FDG PET. A similar article described by Demir et al. (6) in 2007 reported FDG PET and bone scan results in a patient with primary hyperparathyroidism as showing diffusely increased tracer uptake by the skeleton, reflecting metabolic bone disease. However, to the best of our knowledge, superscan secondary to hyperparathyroidism caused by parathyroid carcinoma has not been reported previously and is described herein

**Figure 4:**
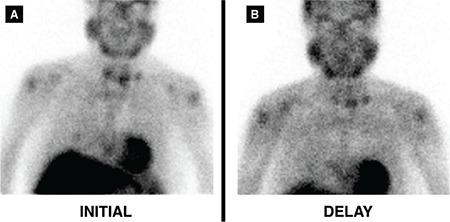
The patient underwent parathyroid scintigraphy for detecting a parathyroid focus, after injection of 740 MBq (20 mCi) MIBI. Three focal MIBI uptakes in the low cervical region were considered as functional parathyroid carcinoma recurrence (A and B). These functional lesions were embolized percutaneously with 95% alcohol under ultrasound guidance in two sessions with an amount of 0.8 cc and 1.5 cc, respectively, by an interventional radiologist experienced in ultrasound guided procedures
